# Oral manifestations associated with Novel Coronavirus Disease - 2019 (COVID-19): A questionnaire based hypothetical study

**DOI:** 10.12688/f1000research.128125.2

**Published:** 2023-05-23

**Authors:** FARAZ MOHAMMED, ARISHIYA THAPASUM FAIROZEKHAN, SHAMAZ MOHAMED, SAUD ABDULLAH ALMOUMEN, AMR S. BUGSHAN, ZAINAB I. ALMOMEN, AMINAH MOHAMMAD ALMOMEN, SHASHI KIRAN M, KHALID S. ALMULHIM

**Affiliations:** 1 Department of Biomedical Dental Sciences, College of Dentistry, Imam Abdulrahman Bin Faisal University, Dammam, P.O. Box 1982, Saudi Arabia; 2 Senior Manager, BioQuest Solutions Pvt Ltd, Bangalore, Karnataka, India; 3 Oral & Maxillofacial Surgery - Dental Division, Ministry of Health, Dammam, Eastern Province, Saudi Arabia; 4 Postgraduate Scholar, Oral & Maxillofacial Surgery, Riyadh Elm University, Riyadh, Saudi Arabia; 5 Medical Intern, College of Medicine, Imam Abdulrahman Bin Faisal University, Dammam, P.O. Box 1982, Saudi Arabia; 6 Medical graduate, Private Practice, Dammam, Eastern Province, Saudi Arabia; 7 Manager, BioQuest Solutions Pvt Ltd, Bangalore, Karnataka, India; 8 Department of Restorative Dental Sciences, College of Dentistry, Imam Abdulrahman Bin Faisal University, Dammam, 31441, Saudi Arabia

**Keywords:** COVID-19, oral symptoms, prevalence, anosmia, ageusia, xerostomia.

## Abstract

**Background:** Since the Coronavirus disease 2019 (COVID-19) outbreak in 2019, the virus has evolved drastically, presenting with sets of mutations that influence its properties, including transmissibility and antigenicity. The oral mucosa is postulated as probable portal entry and several oral manifestations have been identified, which places dental professionals in a position to recognize probable COVID-19 patients depending on oral signs and symptoms in the initial phases of the disease itself. As co-existing with COVID-19 seems to be a new reality, greater understanding is required regarding early oral signs and symptoms which can be predictors for timely intervention and prevention of complications in COVID-19 patients. The objective of the study is to identify the distinguishing oral signs and symptoms among COVID-19 patients and to establish possible correlation between severity of COVID-19 infection and oral symptoms.

**Methods: **This study recruited 179 ambulatory, non-hospitalized COVID-19 patients from the Kingdom of Saudi Arabia’s Eastern Province's designated hotels for COVID-19 and home isolated patients from the same region using a convenience sample method. Data was collected by qualified and experienced investigators, including two physicians and three dentists, using a validated comprehensive questionnaire through telephonic interviews with the participants. The *X ^2^* was used to assess the categorical variables, and odd's ratio was calculated to determine the strength of the association between general symptoms and oral manifestations.

**Results:** Oral and nasopharyngeal lesions or conditions like loss of smell and taste, xerostomia, sore throat, and burning sensation were predictors of COVID-19-related systemic symptoms such as cough, fatigue, fever, and nasal congestion were identified to be statistically significant (p<0.05).

**Conclusions:** The study reveals the occurrence of olfactory or taste dysfunction, dry mouth, sore throat, and burning sensation along with COVID-19 generic symptoms, should be considered as suggestive yet not conclusive indicators of COVID-19.

## Introduction

The outbreak of COVID-19 was followed by a span of evolutionary dormancy. The hallmark of this disease is that it goes undetected for particular period of time, either because it lies dormant or because it is present in just trace amounts. COVID-19 was not detected in nasopharyngeal/sputum samples in many of the instances examined. ^1^ Ever since, COVID-19 has evolved drastically presenting with sets of mutations that influence viral properties, including transmissibility and antigenicity. ^2^ The COVID-19 pandemic has spawned a state of stasis across the world for almost two years. The World Health Organization (WHO), reported 229,373,963 diagnosed cases of COVID-19 with 4,705,111 deaths on September 22, 2021. ^3^

Ample clinical and epidemiological evidence suggests that the COVID-19 virus is extraordinarily virulent, contagious and extensively transmissible among populations by respiratory secretions and via contact and fomites. ^4^ The oral mucosa is thought to be a probable portal of entry for the COVID-19 virus as its cellular entry receptor ACE2 is present in different tissues of oral mucosa, notably in tongue and floor of the mouth. ^5^ With the likelihood of deleterious mutations of COVID-19 virus amidst the new confirmation of effective destruction of some SARS-CoV-2 variants by the newly developed immunizers; it is crucial to have greater understanding of the oral link so that dental professionals can identify potential COVID patients or carriers and provide timely interventions to prevent transmission.

Clinical records have proven that self-reported ageusia and anosmia are strong pointers for the detection of COVID-19 even at a preliminary stage of the disease. ^6^ Various COVID-19-related oral symptoms include xerostomia, mucosal ulcerations, sialadenitis, and periodontal disease apart from gustatory dysfunction. ^7^ The initial COVID-19 symptom of loss of taste, that often precedes fever and or other symptoms, corroborates the hypothesis that perhaps oral cavity, in particularly mucosal membrane of tongue, might be an early niche of viral infection. ^5^

Several underlying mechanisms have been proposed for COVID-19-related oral manifestations. It is possible that dysregulation of the immune system releases inflammatory cytokines that trigger the onset of oral mucosal ulcers. ^7^ Several studies have mentioned different strategies by which SARS-CoV-2 may induce dysgeusia in COVID-19 patients. ^8^ The proposed mechanisms include neural invasion of the virus into gustatory nerves, direct damage of the taste buds by the virus, angiotensin II hormone imbalance, improper sialic acid function, hyposalivation and hypozincemia. ^8^^–^^13^ Direct damage of the salivary glands by the virus, zinc deficiency and inflammatory damage of the glands may cause dry mouth in COVID-19 patients. ^8^^,^^13^ SARS-CoV-2 is evident in saliva of patients with COVID-19 and proven to be detected by salivary reverse transcriptase-polymerase chain reaction (RT-PCR) as it is a more sensitive and reliable testing tool than nasopharyngeal swab test. ^14^

Dental professionals by virtue of the nature of dental practice procedures are at an increased risk of being exposed to body fluids. Close positioning of dental staff to the patients implies that COVID-19 patients or asymptomatic carriers could easily disseminate the disease to dental professionals, and vice versa if appropriate and adequate protective measures are not taken. ^15^

Thus, there are several studies which considered oral transmission to be one of the main routes of COVID‐19 infection, but there is still a gap of knowledge regarding the oral manifestations related to COVID‐19 and its impact on the oral cavity. ^16^ This gap could be due to the lower sample size studied. Therefore, this study was designed to identify the distinguishing oral signs and symptoms in COVID-19 patients and to establish a possible correlation between oral symptoms and gravity of COVID-19 infection by overcoming the previous research gaps.

## Methods

A survey was carried out to determine the prevalence of oral diagnostic features among patients who were diagnosed with COVID-19. The Institutional Review Board of Imam Abdulrahman Bin Faisal University, Dammam, Kingdom of Saudi Arabia approved the study (IRB# 2020-02-220).

Inclusion criteria were based on the diagnostic recommendations for new coronavirus pneumonia (NCIP) of the seventh edition to make sure that the patients included in the research, had positively tested for COVID-19 nucleic acid through the use of RT-PCR or/and next-generation sequencing (NGS) methods before collection of data. ^16^ The study population consisted of ambulatory, non-hospitalized patients who were quarantined in the Kingdom of Saudi Arabia’s Eastern Province’s designated hotels for COVID-19 and those who were home quarantined in the same region. The contact details of the patients were ethically obtained in an anonymous format, without violating the personal privacy of the patients from the authorized COVID-19 testing laboratories’ s Health Electronic Surveillance Network (HESN) database located in the Eastern Province.

A convenience sampling method was adopted for recruiting participants in this study. As it was considerably difficult to get study participants because of the infectious nature of the disease and the social stigma associated with strict COVID-19 protocols, all participants who fulfilled the inclusion requirements and consented were recruited in the study; hence sample size was not taken into consideration. For obtaining consent for participation in the study, a Short Message Service text message (SMS) was sent to all those targeted patients asking them to reply to the same SMS if they were agreeing to involve themselves in the study. Those patients who responded with SMS consent were recruited in the study. Later, one-to-one telephonic interviews were conducted with the respective patients, based on the survey questionnaire, by qualified and experienced investigators including two physicians and three dentists. The study conducted from 21/08/2020 to 07/12/2020.

Data collection was done by means of a comprehensive survey questionnaire which was converted into an online format by using the QuestionPro ^®^ software, to enquire about the systemic and oral manifestation related symptoms of COVID-19 patients. The questionnaire was in accordance with the current literature available about the novel SARS-CoV-2 and COVID-19, including its unique properties, signs and symptoms, recovery after infection, and methods of prevention. The initial component of the survey form included basic demographic details of the participating patients such as age group, gender and type of profession. The latter part of the questionnaire evaluated the COVID-19-related oral and general signs and symptoms. Patients were also asked to report any history of underlying comorbid conditions like diabetes mellitus, hypertension etc. and psychosocial habits including tobacco and substance abuse. The last part of the questionnaire included the questions related to recovery from COVID-19 infection.

The questionnaire was checked for face validity by two independent reviewers. Further, to guarantee the clarity and validity of the questions, the survey form was pilot tested on 15 patients. Based on the responses obtained from the pilot study, certain modifications were made; for the same reason these results were eliminated from the final data which was considered for final analysis. Data collection was based only on telephonic interviews and no clinical examination was performed on any of the study subjects.

### Statistical analysis

The Statistical Package for Social Sciences Software (SPSS V-22, Armonk, NY: IBM Corp) was used to analyse the data. Results were summarised and displayed as frequency distribution tables. The *X ^2^* was used to assess the categorical variables, and odd's ratio was calculated to determine the strength of the association between general symptoms and oral manifestations. Statistical significance was inferred when the p value was ≤ 0.05.

## Results

**Figure 1.**
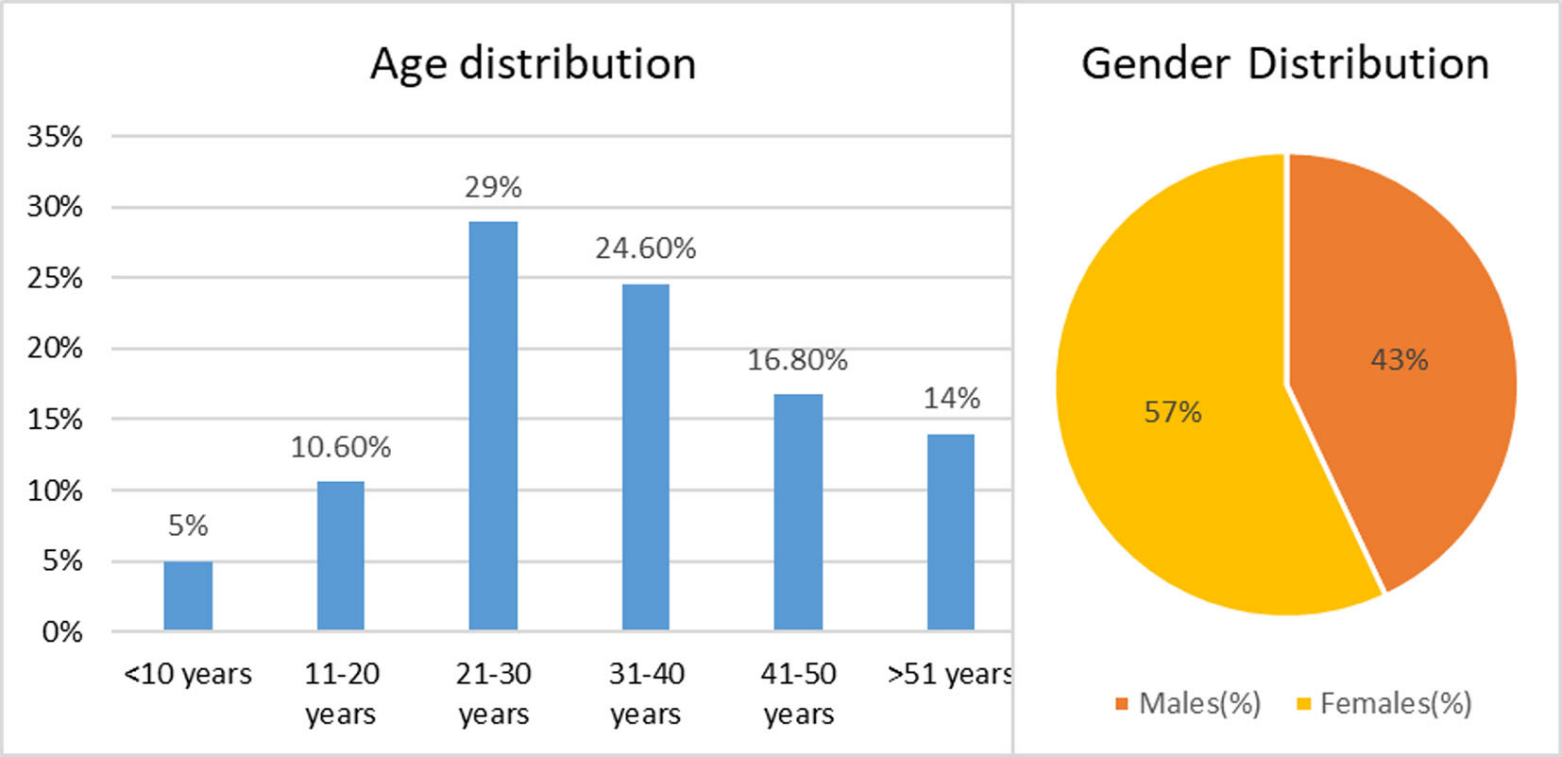
Demographic details of study population.

The current study was designed to obtain insights about the various oral and nasopharyngeal lesions or conditions among patients with history of COVID-19. Participation in this research study was agreed by 230 patients by SMS. Only 179 COVID-19 patients were included for the analysis as few of them did not respond to most questions telephonically. Data analysis was done based on responses obtained from these 179 COVID-19 patients. According to sociodemographic data ( Figure 1), the study population comprised of female patients (57.0%) and male patients (43.0%). The subjects were divided into six age groups, with the largest proportion (29%) belonging to those aged between 21 and 30 years, and the smallest proportion (5.0%) to those aged under 10 years. The vast majority of subjects (82.7%) were not health professionals, with only a handful (17.3%) working in hospitals as COVID-19 warriors.

Considering COVID-19 related symptoms ( Table 1), the order of prevalence in decreasing order were fatigue (75.0%), body pain (74.1%), headache (64.1%), cough (58.7%), fever (55.2%), dizziness (51.5%), subjective fever (48.77%), diarrhea (42.9%), runny nose (37.3%), nasal congestion (32.3%) and shortness of breath (26.1%). Most of the subjects reported that these symptoms had begun before initiation of COVID-19 related treatment. Fever without chills (61.1%) and dry cough (63%) were relatively more common among the study subjects than fever with chills or productive cough.

**Table 1.** Prevalence of COVID-19 related symptoms of the study population.

**Cough (179)**	Yes-105(58.7%)	No-74(41.3%)	
**Type of cough (100)**	Dry cough-63(63%)	Cough with phlegm-37 (37%)	
**Cough had started (98)**	Before the treatment	During the treatment	After the treatment
	84(85.7%)	12(12.2%)	2(2.1%)
**Headache (170)**	Yes -109(64.1%)	No- 61(35.9%)	
**Headache had started (109)**	Before the treatment	During the treatment	After the treatment
	97(89%)	11(10.1%)	1(0.9%)
**Body pain (170)**	Yes-126(74.1%)	No-44(25.9%)	
**Body pain had started (125)**	Before the treatment	During the treatment	
	104(83.2%)	21(16.8%)	
**Fatigue (168)**	Yes-126(75.0%)	No-42(25.0%)	
**Fatigue Started (123)**	Before the treatment	During the treatment	After the treatment
	100(81.3%)	22(17.9%)	1(0.8%)
**Dizziness (163)**	Yes -84(51.5%)	No-79(48.5%)	
**Dizziness started (84)**	Before the treatment	During the treatment	After the treatment
	68(81.0%)	14(16.7%)	2(2.3%)
**Fever (163)**	Yes-90(55.2%)	No-73(44.8%)	
**Type of Fever (90)**	With chills-35(38.9%)	Without chills-55(61.1%)	
**Fever had started (90)**	Before the treatment	During the treatment	
	83(92.2%)	7(7.8%)	
**Subjective fever (162)**	Yes-79(48.77%)	No-83(51.23%)	
**Subjective fever started (79)**	Before the treatment	During the treatment	After the treatment
	54(68.35%)	24(30.38%)	1(1.27%)
**Shortness of breath (161)**	Yes- 42(26.1%)	No-119(73.9%)	
**Shortness of breath started (42)**	Before the treatment	During the treatment	After the treatment
	33(78.6%)	7(16.7%)	2(4.7%)
**Runny Nose (161)**	Yes- 60(37.3%)	No-101(62.7%)	
**Runny Nose started (60)**	Before the treatment-49(81.7%)	During the treatment-11(18.3%)	
**Nasal congestion (161)**	Yes-52(32.3%)	No-109(67.7%)	
**Nasal congestion started (52)**	Before the treatment	During the treatment	
	34(65.4%)	18(34.6%)	
**Diarrhea (161)**	Yes-69(42.9%)	No-92(57.1%)	
**Diarrhea started (69)**	Before the treatment	During the treatment	After the treatment
	48(69.6%)	18(26.1%)	3(4.3%)

**Figure 2.**
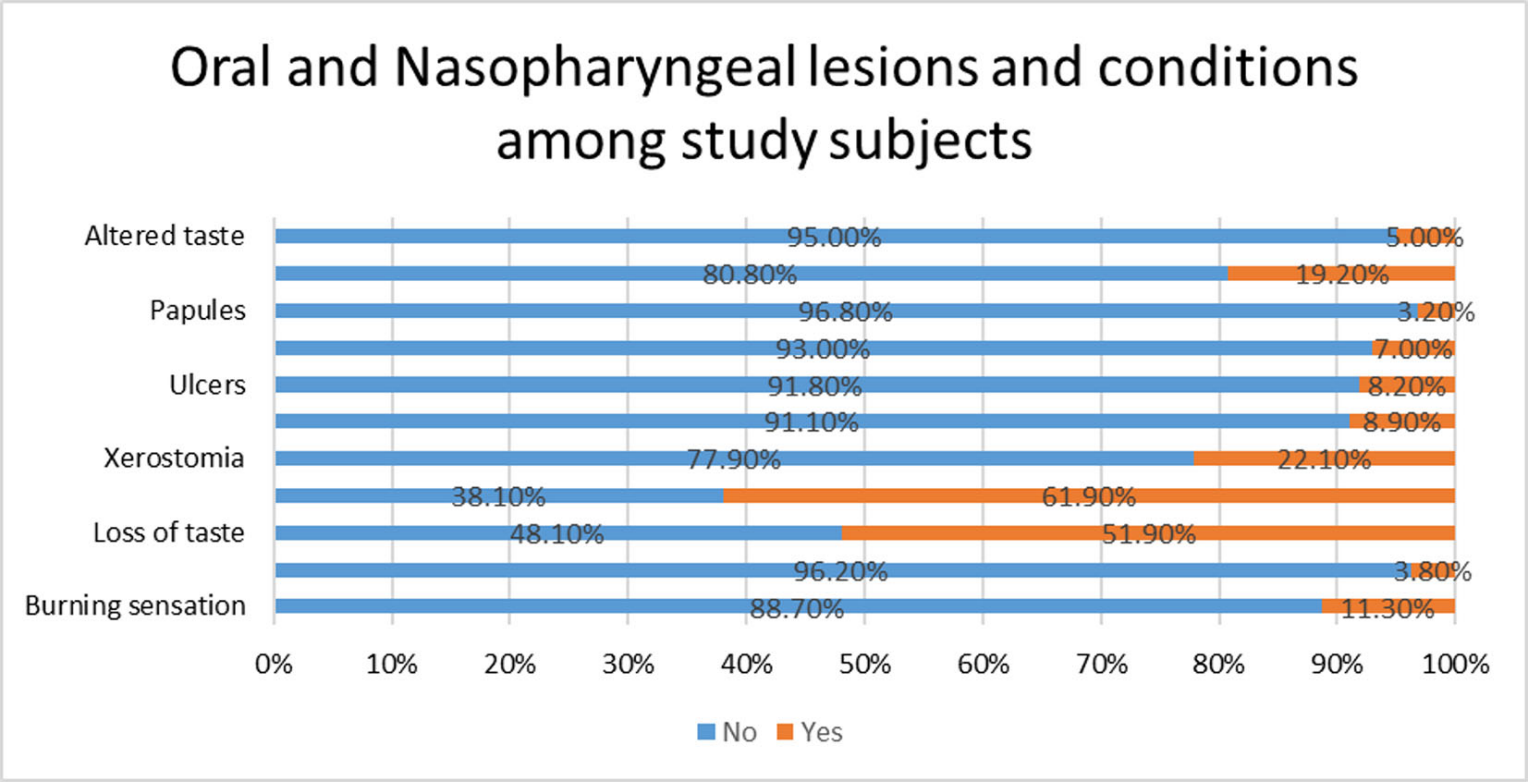
Oral and Nasopharyngeal lesions and conditions among study subjects.

According to frequency distribution of Oral and Nasopharyngeal lesions or conditions ( Figure 2), Loss of smell (61.9%), Loss of taste (51.9%), Xerostomia (22.1%), Sore throat (19.2%), Burning sensation (11.3%), Xerostomia with Burning sensation (8.9%), Ulcers (8.2%), Vesicles (7.0%), Altered taste (5.0%), Tingling sensation (3.8%) and Papules (3.2%) were reported in decreasing order of frequency by study subjects when they were down with COVID-19.

**Figure 3.**
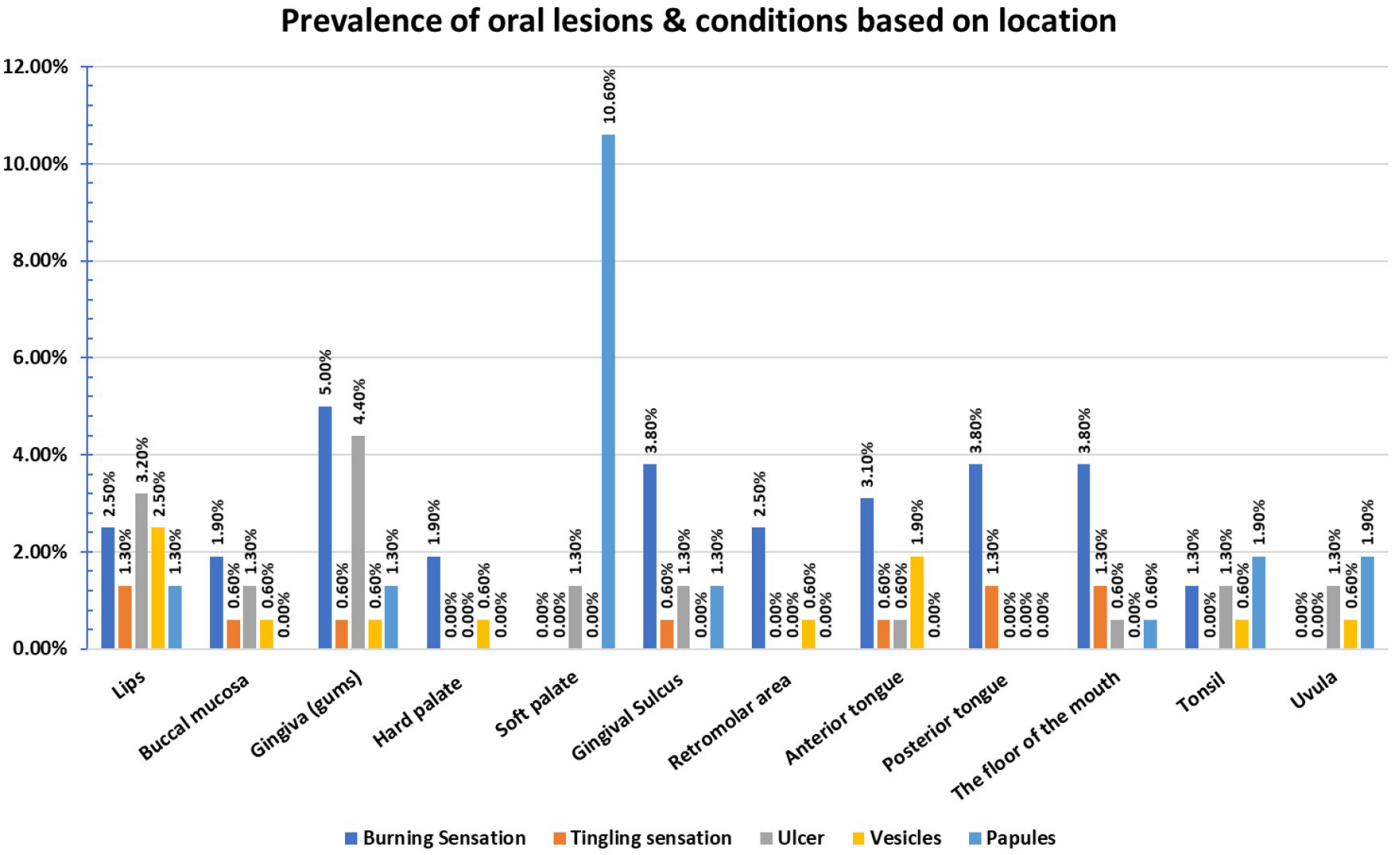
Prevalence of oral lesions and conditions based on location.

Regarding the prevalence of oral lesions and conditions based on location ( Figure 3), Gingiva was found to be the most common site for the occurrence of Burning sensation (5.0%) and ulcers (4.4%). The tingling sensation was most frequently felt on posterior tongue, lips, and floor of the mouth (1.3%). The lips were the most common location for vesicles (2.5%) while the most frequent sites for occurrence of papules were uvula and tonsils (1.9%).

The frequency distribution of oral and nasopharyngeal lesions and conditions associated with COVID-19 in different age groups ( Table 2) revealed statistically significant differences (p0.05) with regard to sore throat, loss of taste and smell. The ‘21 to 30’ and ‘31 to 40’ age groups reported the greatest frequency of loss of taste and smell, followed by the ‘41 to 50’ age group. Loss of smell was least common among children below 10 years of age and none of these children experienced loss of taste. The incidence of sore throat was highest among 41 to 50 years old and lowest among those aged 10 to 20 years age groups.

**Table 2.** Prevalence of COVID-19 related Oral and Nasopharyngeal lesions and conditions among different age groups.

Age group	COVID-19 related Oral and Nasopharyngeal lesions and conditions						
	Loss of smell	Loss of taste	Xerostomia	Sore throat	Burning sensation	Ulcers	Vesicles
**Below 10 years**	1(11.1%)	0(0.0%)	2(22.2%)	2(22.2%)	1(11.1%)	1(11.1%)	0(0.0%)
**10 to 20 years**	7(38.9%)	7(38.9%)	3(16.7%)	1(5.6%)	1(5.6%)	0(0.0%)	1(5.6%)
**21 to 30 years**	37(75.5%)	30(61.2%)	11(23.4%)	8(17.0%)	6(12.2%)	4(8.5%)	4(8.5%)
**31 to 40 years**	27(75.0%)	22(61.1%)	5(13.9%)	5(14.3%)	1(2.8%)	4(11.1%)	3(8.6%)
**41 years to 50 years**	19(65.5%)	17(58.6%)	9(31.0%)	10(34.5%)	6(20.7%)	3(10.3%)	1(3.4%)
**51 years and above**	8(42.1%)	7(36.8%)	5(26.3%)	4(22.2%)	3(15.8%)	1(5.3%)	2(10.5%)
p value*	<0.001**	0.007*	0.65	0.03*	0.28	0.77	0.85

p values based on Chi-square test; considered statistically significant at p≤0.05

Gender based comparison for occurrence of Oral and Nasopharyngeal lesions or conditions ( Table 3) showed that Anosmia, Ageusia, Xerostomia, Sore throat, Burning sensation and Vesicles were more commonly reported by females while males had a higher prevalence of ulcers; however these differences were statistically insignificant (p>0.05).

Odds’ ratios showing strength of association ( Table 4) showed that Oral/Nasopharyngeal lesions or conditions like Anosmia, Ageusia, Xerostomia, Sore throat and Burning Sensation can be predictors of COVID-19 related systemic symptoms like cough, fatigue, subjective fever and nasal congestion were found to be statistically significant (p≤0.05). Anosmia, Ageusia, Sore throat and Burning Sensation showed statistically significant (p≤0.05) association with symptoms of Body pain and Shortness of Breath. The strongest predictor was observed for occurrence of burning sensation and body pain (OR=16.18) which indicates that there was 16 times more chances of burning sensation to be reported among those with COVID-19 related body pain than without. Statistically significant association(p≤0.05) of symptoms like fever, runny nose and Diarrhea were limited only to oral findings like xerostomia, burning sensation and sore throat respectively.

**Table 3.** Prevalence of COVID-19 related Oral and Nasopharyngeal lesions and conditions among Males and Females.

Gender	COVID-19 related Oral and Nasopharyngeal lesions and conditions						
	Loss of smell	Loss of taste	Xerostomia	Sore throat	Burning sensation	Ulcers	Vesicles
**Males**	42(56.8%)	37(50.0%)	7(36.8%)	13(17.6%)	6(8.1%)	7(9.5%)	4(5.4%)
**Females**	57(66.3%)	46(53.5%)	7(43.8%)	17(20.7%)	12(14.0%)	6(7.1%)	7(8.4%)
p value*	0.14	0.38	0.47	0.38	0.18	0.41	0.33

p values based on Chi-square test; considered statistically significant at p≤0.05

**Table 4.** Odds’ ratios showing 95% confidence interval and strength of association between Oral/Nasopharyngeal lesions or conditions and Covid-19 related systemic symptoms.

Covid-19 symptoms	Odd’s ratios				
	Loss of smell	Loss of taste	Xerostomia	Sore throat	Burning sensation
**Cough**	2.1095 (95% CI, 1.100±4.047)	4.6742 (95% CI, 2.371±9.213)	2.8209 (95% CI, 1.188±6.700)	2.7143 (95% CI, 1.086±6.783)	3.99 (95% CI, 1.105±14.385)
**p value**	**0.0247**	**0.0001**	**0.0188**	**0.0326**	**0.0346**
**Headache**	2.7556 (95% CI, 1.411±5.380)	3.0692 (95% CI, 1.566±6.015)	1.8489 (95% CI, 0.798±4.283)	6.5342 (95% CI, 1.883±22.673)	2.1477 (95% CI, 0.672±6.863)
**p value**	**0.0030**	**0.0011**	0.1516	**0.0031**	0.1972
**Body pain**	3.1250 (95% CI, 1.518±6.433)	4.6545 (95% CI, 2.134±10.151)	2.4828 (95% CI, 0.892±6.908)	6.2759 (95% CI, 1.424±27.664)	16.1759 (95% CI, 11.311±20.113)
**p value**	**0.0020**	**0.0001**	0.0816	**0.0152**	**0.0540**
**Fatigue**	3.6024 (95% CI, 1.720±7.546)	4.1779 (95% CI, 1.910±9.137)	3.3343 (95% CI, 1.099±10.121)	3.8864 (95% CI, 1.111±13.592)	15.13 (95% CI, 9.463±20.514)
**p value**	**0.0007**	**0.0003**	**0.0335**	**0.0336**	**0.05**
**Dizziness**	2.0371 (95% CI, 1.065±3.895)	1.8227 (95% CI, 0.973±3.414)	1.4758 (95% CI, 0.688±3.165)	3.7312 (95% CI, 1.494±9.321)	2.0000 (95% CI, 0.711±5.623)
**p value**	**0.0315**	0.0608	0.3175	**0.0048**	0.1888
**Fever**	0.6619 (95% CI, 0.346±1.265)	1.0916 (95% CI, 0.586±2.035)	2.4597 (95% CI, 1.090±5.552)	1.8769 (95% CI, 0.814±4.329)	1.3647 (95% CI, 0.500±3.722)
**p value**	0.2115	0.7826	**0.0302**	0.1397	0.5436
**Subjective Fever**	3.9819 (95% CI, 1.998±7.937)	3.4890 (95% CI, 1.817±6.698)	2.4491 (95% CI, 1.119±5.362)	7.6000 (95% CI, 2.729±21.168)	10.6230 (95% CI, 2.354±47.946)
**p value**	**0.0001**	**0.0002**	**0.0251**	**0.0001**	**0.0021**
**Shortness of breath**	2.7595 (95% CI, 1.211±6.287)	3.9942 (95% CI, 1.796±8.885)	2.0049 (95% CI, 0.897±4.483)	3.3654 (95% CI, 1.457±7.772)	4.4758 (95% CI, 1.628±12.306)
**p value**	**0.0157**	**0.0007**	0.0903	**0.0045**	**0.0037**
**Runny nose**	1.7602 (95% CI, 0.892±3.472)	1.3611 (95% CI, 0.715±2.591)	1.6241 (95% CI, 0.758±3.481)	2.0732 (95% CI, 0.925±4.646)	2.9825 (95% CI, 1.088±8.179)
**p value**	0.1029	0.3480	0.2125	0.0766	**0.0337**
**Nasal congestion**	5.3000 (95% CI, 2.282±12.308)	2.9202 (95% CI, 1.449±5.887)	2.1173 (95% CI, 0.979±4.581)	6.6897 (95% CI, 2.817±15.889)	5.1000 (95% CI, 1.792±14.515)
**p value**	**0.0001**	**0.0027**	**0.0548**	**0.0001**	**0.0023**
**Diarrhea**	0.9919 (95% CI, 0.520±1.891)	1.0771 (95% CI, 0.575±2.018)	1.5459 (95% CI, 0.727±3.286)	4.0580 (95% CI, 1.716±9.599)	1.8103 (95% CI, 0.674±4.863)
**p value**	0.9803	0.8165	0.2576	**0.0014**	0.2391

## Discussion

The disastrous impacts of the widespread COVID-19 pandemic on all sectors has detrimentally afflicted the quality of life globally. Though two years have elapsed, coexisting with Covid-19 seems to have become the ‘New normal’ as the world has recognized and accepted the reality that Covid-19 and its mutant variants are here to stay for quite a long time. ^17^

With the development and easy availability of COVID-19 vaccines, most countries are now relaxing the restrictions for wearing masks and socializing as an attempt to resolve the economic, social and medical burden which are resultant aftermath of the pandemic. So, it is quite normal for undiagnosed COVID-19 patients or carriers to spend time with healthy individuals during social or official gatherings, which can initiate waves of fresh infection. For the same reason the dental fraternity should be cautious about infectious carriers of COVID-19 who might present themselves for routine dental treatment procedures. It is said that oral health mirrors general health; surprisingly till date there is only scanty data related to the prevalence of oral lesions and its association with other COVID-19 related symptoms. Much of the available literature in this field is in the form of case reports, case series and systematic reviews making completely valid and meaningful comparisons with other similar observational studies a difficult challenge.

In the present study oral symptoms were significantly associated with many of the COVID-19 related general symptoms showing that these could be suitable predictors for confirmation of COVID-19 without waiting for diagnostic test reports.

Anosmia, sore throat, ageusia, burning sensation and xerostomia were among the most prevalent Oral and Nasopharyngeal lesions or conditions reported by the respondents of the present study. Orilisi *et al.* in their systematic review mentioned the occurrence of functional disorders like xerostomia, ageusia, dysgeusia and burning mouth as early manifestations of hospitalized patients affected by COVID-19 infection. ^18^ This is in accordance with the findings of current study where these commonly reported symptoms had begun before initiation of COVID-19 related treatment. This indicates that these findings can be attributed as distinguishing features of COVID -19 and not as any side effects of drugs used for COVID-19 treatment.

According to the present study, Anosmia and Ageusia, were reported by 61.9% and 51.9% of the study subjects respectively. This is similar to the conclusions made by Mullol *et al.*, in their review article stating that Smell impairment is frequent and presents as an early and abruptly occurring distinguishing symptom in COVID-19 in at least 1 out of 5 patients. ^19^ Anosmia and Ageusia, were the most reported differentiating symptoms among COVID-19 patients in studies by Kumar *et al.* ^20^ Ageusia was a dominant symptom noted in other observational studies by Ganesan *et al.*, (51.2%) ^21^, Elkady *et al.*, (34.5%) ^22^ and Natto *et al.*, (43.4%) ^23^ The incidence of smell and/or taste disorders ranged from 5% to 98%, based upon on region of the study and study design. ^19^ There is substantial evidence that loss of Anosmia or Ageusia is strongly linked with COVID-19 and can be used as questions to screen the patients in medicine and dentistry clinics to limit the risk of disease transmission. ^24^

Xerostomia was yet another prominently reported symptom in this study, with a prevalence of 22.1% which was consistent with the findings of Ganesan *et al.*, ^21^ and Elkady *et al.*, ^22^. However, in a case series by Fathi *et al.*, 60% of cases gave history of dry mouth, 3–4 days prior as a prodromal symptom which was not in agreement to our results. ^25^ Soares *et al.*, detected COVID-19 virus in the vacuolated cells of the superficial epithelium and also in the salivary glands of COVID-19 patients, indicating that salivary glands can be considered as a viral pool and saliva may possibly be the main source of the contagion. ^26^

Sore throat was prevalent among 19.2% of current respondents which did not tally with studies by Savtale *et al.*, (47.2%) ^27^, or Alsofayan *et al.*, (81.6%) ^28^ but was in close alignment with reports by Al-Omari *et al.*, (21.9%) ^29^ and Biadsee *et al.*, (26.6%) ^30^. The difference could have been due to difference in age groups, gender or presence of other comorbidities among the study subjects.

In the current study, ulcers, vesicles, and papules were also more or less frequently found findings. Such lesions might result from different conditions like infections, poor oral hygiene, immunosuppression states, trauma or neoplasms. ^18^^,^^31^ An elevated level of tumor necrosis factor (TNF)- α in individuals with COVID-19 can lead to chemotaxis of neutrophils to the oral mucosa and thus growth of aphthous-like lesions. Other plausible reasons for the formation of such lesions in these patients include concomitant stress and immunosuppression brought on by the COVID-19 infection. ^14^ These oral lesions thereby cannot be considered as COVID-19 specific manifestations.

General systemic symptoms like Fatigue, Body pain, Headache, Cough, Fever, Dizziness, Subjective fever, Diarrhea, Runny nose, Nasal congestion, and Shortness of Breath were commonly prevalent among our study subjects which were similar to observations by Al-Omari *et al.*, ^29^ and Rothan *et al.* ^32^

Based on calculated odds ratios for the present study which showed statistically significant association, Anosmia was twice more likely to be present among those with cough and dizziness, thrice more common among those with headache, body pain and shortness of breath, four times commonly associated with subjective fever and five times with nasal congestion; whereas loss of taste was thrice more likely to occur in subjects with headache, four times with fatigue, subjective fever and shortness of breath and five times more likely to be found among those with cough and body pain. According to Kumar *et al.*, ^20^ the association between olfactory or gustatory impairment and fever was substantial and favourable (Odds ratio = 10.60) which was higher than OR≈4 in our study; positive association was also reported with diarrhea (Odds ratio = 4.86); however, no significant association was detected for loss of taste or smell with occurrence of diarrhea in the present study. The difference in the age groups considered for both studies could be a possible explanation for the differences in odds ratios observed. The current study also showed OR≈3, associating xerostomia with fatigue, fever and cough and OR≈2 for association with nasal congestion but no possible comparative studies were available associating xerostomia, sore throat or burning sensation with COVID-19 general symptoms.

## Conclusion & limitations

The results of this study indicate that the presence of olfactory (smell) or gustatory (taste) dysfunction, dry mouth, sore throat, and a burning sensation, along with COVID-19 generic symptoms, should be regarded as suggestive but not definite markers of COVID-19. In spite of the fact that the research was only conducted in Eastern Province, it was able to identify and include the very first cases of COVID-19 in the Eastern Province of the Kingdom of Saudi Arabia. In addition to this, the social factors of the patients were accounted for, and the sample size was adequate, covering a broad spectrum of clinical data. It was unable to fully analyse the clinical data for some patients because there was insufficient information available regarding the frequency and duration of these patients' self-reported symptoms. Further research is definitely required and should include parameters like viral load, quantitative assessment of symptoms and evaluation of histopathological parameters to confirm the extent of cause effect relation between oral and systemic findings of COVID-19. With detailed oral screening, dental professionals are in an optimal position to take adequate precautions for prevention and timely intervention for early diagnosis and prompt treatment to avoid potential complications and community spread.

## Data Availability

Figshare: Data set - Oral manifestations associated with Novel Coronavirus Disease - 2019 (COVID-19): A questionnaire based hypothetical study, https://doi.org/10.6084/m9.figshare.21546324.v1. ^33^ Figshare: Questionnaire - Oral manifestations associated with Novel Coronavirus Disease - 2019 (COVID -19): A questionnaire based hypothetical study, https://doi.org/10.6084/m9.figshare.21528999.v1. ^34^ Data are available under the terms of the Creative Commons Attribution 4.0 International license (CC-BY 4.0).
