# Supplementary material for: Oral manifestations associated with Novel Coronavirus Disease - 2019 (COVID-19): A questionnaire based hypothetical study
Source: F1000Res. 2023 May 23;11:1443. Originally published 2022 Dec 6. [Version 2] doi: 10.12688/f1000research.128125.2 (PMC10285348; doi:10.12688/f1000research.128125.2)
Supplement: Peer review history

# Peer review: Reviewer response for version 2

**DOI:** 10.5256/f1000research.147248.r174712

**Published:** 2023-06-13

**Authors:** Muhammad Amber Fareed

**Affiliations:** 1 College of Dentistry, Gulf Medical University, Ajman, United Arab Emirates

I’m satisfied with the revisions made by the authors and happy to recommend for this manuscript for indexing.

Is the work clearly and accurately presented and does it cite the current literature?

Yes

If applicable, is the statistical analysis and its interpretation appropriate?

I cannot comment. A qualified statistician is required.

Are all the source data underlying the results available to ensure full reproducibility?

Yes

Is the study design appropriate and is the work technically sound?

Yes

Are the conclusions drawn adequately supported by the results?

No

Are sufficient details of methods and analysis provided to allow replication by others?

Yes

Reviewer Expertise:

NA

I confirm that I have read this submission and believe that I have an appropriate level of expertise to confirm that it is of an acceptable scientific standard.

# Peer review: Reviewer response for version 2

**DOI:** 10.5256/f1000research.147248.r174713

**Published:** 2023-05-25

**Authors:** Shruthi Acharya

**Affiliations:** 1 Department of Oral Medicine and Radiology, Manipal College of Dental Sciences, Manipal Academy of Higher Education, Manipal, Karnataka, India

I appreciate the efforts put in by the authors in addressing the concerns raised.

Is the work clearly and accurately presented and does it cite the current literature?

Yes

If applicable, is the statistical analysis and its interpretation appropriate?

Yes

Are all the source data underlying the results available to ensure full reproducibility?

Yes

Is the study design appropriate and is the work technically sound?

Yes

Are the conclusions drawn adequately supported by the results?

Yes

Are sufficient details of methods and analysis provided to allow replication by others?

Yes

Reviewer Expertise:

Oral Cancer Diagnosis, Potentially malignant oral disorders, CBCT, Oral mucosal lesions, Management of oral complications of cancer therapy

I confirm that I have read this submission and believe that I have an appropriate level of expertise to confirm that it is of an acceptable scientific standard.

# Peer review: Reviewer response for version 1

**DOI:** 10.5256/f1000research.140687.r160633

**Published:** 2023-02-01

**Authors:** Shruthi Acharya

**Affiliations:** 1 Department of Oral Medicine and Radiology, Manipal College of Dental Sciences, Manipal Academy of Higher Education, Manipal, Karnataka, India

I congratulate the authors for performing the study to understand the oral manifestations of COVID-19 infection. The study was done under challenging circumstances. I appreciate the efforts put in by the authors to conduct the study. 

 I have a few queries:

1. Kindly justify why the term “hypothetical” is used in the title to describe the study
2. Introduction - well written
3. Patients and Methods - IEC approval was obtained, Inclusion criteria is defined and the questionnaire was vetted.
4. Results 
  1. Kindly rephrase and reorder sentences in the first paragraph of the results section.
  2. Children less than 10 years were included in the study - were they able to comprehend and explain their symptoms like papule/ vesicle, etc. over the telephone?
  3. Kindly use uniform spelling for “Diarrhea” in the text and table.
  4. Table 1 - kindly restructure the table. Symptoms with Yes/No response to be put together, Symptoms with Before the treatment/During the treatment/ after treatment responses together.
  5. Figure 3-  The text and legends are not visible clearly. Kindly improve the resolution of the figure.
  6. Kindly do not unnecessarily capitalize words between a sentence.
  7. Table 4 - kindly provide reference categories and 95% confidence intervals for all the variables. Also, provide legends to the tables.
5. Discussion
  1. Kindly do not unnecessarily capitalize words between a sentence.
6. Drawbacks of study
  1. It can be described as a separate heading.
  2. Is there a possibility of recall bias?
7. Conclusion can be clearly stated.
8. References
  1. Some references are not complete.
  2. References should have both start and end page numbers.

Is the work clearly and accurately presented and does it cite the current literature?

Yes

If applicable, is the statistical analysis and its interpretation appropriate?

Yes

Are all the source data underlying the results available to ensure full reproducibility?

Yes

Is the study design appropriate and is the work technically sound?

Yes

Are the conclusions drawn adequately supported by the results?

Yes

Are sufficient details of methods and analysis provided to allow replication by others?

Yes

Reviewer Expertise:

Oral Cancer Diagnosis, Potentially malignant oral disorders, CBCT, Oral mucosal lesions, Management of oral complications of cancer therapy

I confirm that I have read this submission and believe that I have an appropriate level of expertise to confirm that it is of an acceptable scientific standard, however I have significant reservations, as outlined above.

# Response

**Published:** 2023-05-10

**Authors:** FARAZ MOHAMMED

**Affiliations:** College of Dentistry, Imam Abdulrahman Bin Faisal University, Dammam, Saudi Arabia

**Reviewer comment: **Kindly justify why the term “hypothetical” is used in the title to describe the study.

**Authors Reply:**  The term hypothetical was used in the title because, this study was conducted in the very early phase of COVID-19 era. At that particular time no data was published on the oral manifestations associated with COVID-19 except for loss of taste. Since we knew few oral signs & symptoms of few other viral infections, we took this study as a hypothetical study.

**Reviewer comment: **Introduction - well written

**Authors Reply:**  Thank you so much

**Reviewer comment: **Kindly rephrase and reorder sentences in the first paragraph of the results section.

**Authors Reply:**  Thank you so much for the constructive comment. We have now rephrased and reordered sentences in the first paragraph of the results section.

**Reviewer comment: **Children less than 10 years were included in the study - were they able to comprehend and explain their symptoms like papule/ vesicle, etc. over the telephone?

**Authors Reply:**  Since the questionnaire was drafted both in English and Arabic languages. Moreover, the doctors who interviewed these patients were well versed in the native language and they could clearly explain to the patient. At times we took the help of the parents. 

**Reviewer comment: **Kindly use uniform spelling for “Diarrhea” in the text and table.

**Authors Reply:** Sorry, we have now used the uniform spelling.

**Reviewer comment: **Kindly do not unnecessarily capitalize words between a sentence.

**Authors Reply:** Sorry, we have now removed the unnecessarily capitalize words from between a sentence.

**Reviewer comment: **Table 4 - kindly provide reference categories and 95% confidence intervals for all the variables. Also, provide legends to the tables.

**Authors Reply: **We have now provided 95% confidence intervals for all the variables. The legends to the table are also provided.

**Discussion**

**Reviewer comment: **Kindly do not unnecessarily capitalize words between a sentence.

**Authors Reply:** Sorry, we have now removed the unnecessarily capitalize words from between a sentence.

**Drawbacks of study**

**Reviewer comment: **It can be described as a separate heading.

**Authors Reply:** Yes, we have now included a separate paragraph as “Conclusion & Limitations”.

**Reviewer comment: **Is there a possibility of recall bias?

**Authors Reply: **This study was conducted in the very early phase of COVID-19 where the patients had just recovered from COVID-19 infection, thereby there was very less chances of patients forgetting a past events of their infection. Moreover, 230 patients participated in this study. But, only 179 COVID-19 patients were included for the statistical analysis as few of them did not respond to most questions. So completely elimination the recall bias.

**Conclusion**

**Reviewer comment: **Conclusion can be clearly stated.

**Authors Reply:** Yes, we have now included a separate paragraph as “Conclusion”.

**References**

**Reviewer comment: **Some references are not complete.

**Authors Reply: **Now we have updated the references according to the journal norms. Most of the articles from the MDPI journals have a single page number. And the references are automatically generated according to journal norms.

**Reviewer comment: **References should have both start and end page numbers.

**Authors Reply: **Now we have updated the references according to the journal norms.

# Peer review: Reviewer response for version 1

**DOI:** 10.5256/f1000research.140687.r157555

**Published:** 2023-01-16

**Authors:** Muhammad Amber Fareed

**Affiliations:** 1 College of Dentistry, Gulf Medical University, Ajman, United Arab Emirates

I’ve reviewed the manuscript titled “Oral manifestations associated with Novel Coronavirus Disease - 2019 (COVID-19): A questionnaire-based hypothetical study” with interest. The authors of this study presented data on the correlation between the severity of COVID-19 infection and oral symptoms while distinguishing oral signs and symptoms among ambulatory and non-hospitalized COVID-19 patients presented in Saudi Arabia during the COVID-19 pandemic outbreak in 2020. This is useful and relevant research in relation to the most recent pandemic condition of the COVID-19 pandemic. The finding of this study certainly provides some recommendations and supports health workers to better understand COVID-19 manifestation of oral conditions.

 Please, find my following comments and questions about this manuscript:

- The title and abstract look ok as they cover the main idea and condense the key findings.
- Introduction: The authors have provided a brief over and introduction to the topic and have highlighted several underlying mechanisms for COVID-19-related oral manifestations. It would be interesting if the authors may clarify and include a similar study using the patient interview and may comment on whether earlier research had some limitations and use these gaps to explain the novelty of this research. Please also provide any relevant recent references on the same topic from Saudi Arabia to explain whether similar studies that reported the oral manifestations associated with coronavirus disease.
- Method: Please provide more details on how the sample size was calculated. 
- Discussion: Authors have provided a reasonable argument to support the finding of this study however, I would encourage authors to discuss the finding of this work in relation to the case reports published including the parameters on viral load, quantitative assessment of symptoms and evaluation of histopathological parameters.
- The conclusions are missing.
- It would a good idea to provide the “comprehensive survey questionnaire” as additional resource.

Is the work clearly and accurately presented and does it cite the current literature?

Yes

If applicable, is the statistical analysis and its interpretation appropriate?

I cannot comment. A qualified statistician is required.

Are all the source data underlying the results available to ensure full reproducibility?

Yes

Is the study design appropriate and is the work technically sound?

Yes

Are the conclusions drawn adequately supported by the results?

No

Are sufficient details of methods and analysis provided to allow replication by others?

Yes

Reviewer Expertise:

Dental biomaterials and restorative dentistry research

I confirm that I have read this submission and believe that I have an appropriate level of expertise to confirm that it is of an acceptable scientific standard, however I have significant reservations, as outlined above.

# Response

**Published:** 2023-05-10

**Authors:** FARAZ MOHAMMED

**Affiliations:** College of Dentistry, Imam Abdulrahman Bin Faisal University, Dammam, Saudi Arabia

**ABSTRACT**

**Reviewer comment:** The title and abstract look ok as they cover the main idea and condense the key findings.

**Authors Reply:**  Thank you so much for your constructive comment.

**Introduction:**

**Reviewer comment:** The authors have provided a brief over and introduction to the topic and have highlighted several underlying mechanisms for COVID-19-related oral manifestations. It would be interesting if the authors may clarify and ***include a similar study using the patient interview and may comment on whether earlier research had some limitations and use these gaps to explain the novelty of this research.*** Please also provide any relevant recent references on the same topic from Saudi Arabia to explain whether similar studies that reported the oral manifestations associated with coronavirus disease.

**Authors Reply:**  The authors have now included a similar study using the patient interview and also have commented on the limitations and gaps from the previous studies. ****As far as the other concern of the esteemed reviewer to include a similar study from Saudi Arabia, we the authors like to inform our reviewer that, our study was the very first study to be carried out in the Kingdom of Saudi Arabia during the period 21/08/2020 to 07/12/2020, further that no similar studies were published from the Kingdom of Saudi Arabia when we first submitted our manuscript for publication.

**Method:**

**Reviewer comment:** ***Please provide more details on how the sample size was calculated. - Given in the result section.***

**Authors Reply:**  A convenience sampling method was adopted for recruiting participants in this study. As it was considerably difficult to get study participants because of the infectious nature of the disease and the social stigma associated with strict COVID-19 protocols, all participants who fulfilled the inclusion requirements and consented were recruited in the study; hence sample size was not taken into consideration. *This statement is been included in the manuscript.*

**Discussion:**

**Reviewer comment:** Authors have provided a reasonable argument to support the finding of this study however, I would encourage authors to discuss the finding of this work in relation to the case reports published including the parameters on viral load, quantitative assessment of symptoms and evaluation of histopathological parameters.

**Authors Reply:**  Thank you so much for your constructive comment on the written discussion. We have already included the case reports and correlated and discussed the findings elaborately in the manuscript. But for the comment to include and discuss the evaluation of histopathological parameters, we the authors have not taken any biopsy from the studied samples due to the fact considerably difficult to get study participants because of the infectious nature of the disease and the social stigma associated with strict COVID-19 protocols. So we could not discuss this aspect in our manuscript.

**Conclusion:**

**Reviewer comment:** The conclusions are missing.

**Authors Reply:**  Earlier the conclusion was merged in the discussion paragraph itself. Now the conclusion and limitations part are separated from the discussion part and added separately in the manuscript.

**Reviewer comment:** It would a good idea to provide the “comprehensive survey questionnaire” as additional resource.

**Authors Reply:**  Yes, the authors have already provided and also deposited the “comprehensive survey questionnaire” as well as the complete “data set” in Repository with the following hyperlinks:

**Data availability**

***Underlying data***

 Figshare: Data set - Oral manifestations associated with Novel Coronavirus Disease - 2019 (COVID-19): A questionnaire based hypothetical study, https://doi.org/10.6084/m9.figshare.21546324.v1

***Extended data***

 Figshare: Questionnaire - Oral manifestations associated with Novel Coronavirus Disease - 2019 (COVID -19): A questionnaire based hypothetical study, https://doi.org/10.6084/m9.figshare.21528999.v1

 Data are available under the terms of the Creative Commons Attribution 4.0 International license (CC-BY 4.0).
